# Performance Investigation and Cost–Benefit Analysis of Recycled Tire Polymer Fiber-Reinforced Cemented Paste Backfill

**DOI:** 10.3390/polym14040708

**Published:** 2022-02-12

**Authors:** Zhuoqun Yu, Yongyan Wang, Jianguang Li

**Affiliations:** School of Electromechanical Engineering, Qingdao University of Science and Technology, Songling Road No. 99, Qingdao 266061, China; yzqun2007@126.com (Z.Y.); 02545@qust.edu.cn (J.L.)

**Keywords:** polymer fibers, cemented paste backfill materials, scrap tires

## Abstract

To alleviate the environmental problems caused by scrap tire and tailings disposal, the performance of recycled tire polymer fiber (RTPF)-reinforced cemented paste backfill (CPB) was investigated. Ordinary CPB, commercial poly-propylene fiber (CPPF) and reinforced CPB were also investigated for comparison. Slump tests, unconfined compression tests and a cost–benefit analysis were conducted. The results indicate that the flowability of the RTPF-reinforced CPB decreased with the increasing fiber content. The failure strain, unconfined compressive strength, and toughness values were generally higher than that of ordinary CPB (i.e., CPB without any fiber reinforcement). However, the mechanical properties would not be improved continuously with increasing RTPF content. It was found that the inclusion of RTPFs achieved the best improvement effect with the best mechanical properties of CPB at the fiber content of 0.6%. The failure mode of the RTPF-reinforced CPB was safer than that of the ordinary CPB. Microscopic observations indicated that the bond between RTPFs and the CPB matrix could affect the mechanical properties of the RTPF-reinforced CPB. From the cost–benefit analysis, the inclusion of RTPFs to reinforce CPB could gain relatively high mechanical properties with a low material cost.

## 1. Introduction

The consumption of non-renewable natural materials and energy in the material manufacturing industry is becoming humongous around the world, which may cause huge waste production and environmental pollution. Worse still, this trend is expected to continue growing due to economic development and population growth. From the literature, more than 3 billion metric tons of virgin materials are consumed worldwide annually [1]. It can be estimated that the material and energy consumption globally will increase by 300% over the next 50 years [2]. On the other hand, the disposal of solid waste has become a serious challenge. Scrap tire is a common and abundant solid waste that is generated in the transportation industry around the world continually. However, the improper disposal of scrap tires causes serious adverse environmental impacts [3]. For instance, several fire accidents at the stockpiled scrap tires happened during the past 30 years [4,5,6]. The hazardous air contaminants such as benzene, dioxins and polychlorinated biphenyls generated from the burning tires could result in the severe pollution of air, water and soil [7]. Since the reuse of solid wastes and recycled materials could reduce raw material and energy consumption [8], a general strategy of recycling raw material applications must be developed for preserving raw materials with reduced environmental impact.

Typically cemented paste backfill (CPB) is an environmentally friendly material consisting of tailings, cement and water, which can be widely used in underground mining operations for preventing caving, roof falls and enhancing the recovery of pillars [9,10,11]. The application of CPB technology provides an attractive proposition to solve the waste management problem of tailings [12]. Mechanical stability at low binder cost is one of the most important requirements for CPB structures [13,14].

In recent years, some researchers have used virgin materials, such as commercial poly-propylene fiber (CPPF), to prepare fiber-reinforced CPB. It is generally believed that CPPFs can enhance ductility and provide resisting forces for CPB. For instance, Yi et al. [15] reported that CPPF-reinforced CPB had a higher unconfined compressive strength (UCS) than ordinary CPB (i.e., CPB without any reinforcement). Chen et al. [16] also reported similar findings with increased failure strain. Although these results show the great improvement effect of CPPF on the mechanical properties of CPB, the material cost is an issue that needs to be addressed. Besides, the extensive use of virgin commercial materials on the reinforcement of CPB will greatly increase the consumption of natural resources, including raw materials and energy. 

Extensive studies have been carried out to research the feasibility of using recycled materials from scrap tires to reinforce CPB or other cementitious composites due to the increasing need for fiber-reinforced materials with low costs and less environmental impact [17,18,19]. For instance, Wang et al. [19] added rubber fibers into CPB to modify the mechanical behavior of CPB. Yang et al. [20] reported that crumb rubber can be mixed into petroleum asphalt for performance improvement. In the process, a considerable amount of recycled tire polymer fiber (RTPF) was produced, which was one of the byproducts derived from the crumb rubber production process. 

The feasibility of using RTPFs as construction materials has been studied in recent years. Onuaguluchi et al. [21] added RTPFs into concrete to enhance the plastic shrinkage crack resistance with an increased residual load-bearing capacity of the cement mixtures. Figueiredo et al. [22] found that the inclusion of RTPFs into concrete has the potential to prevent fire spalling. These researchers have shown the great potential for the application of RTPFs in the reinforcement of construction materials. 

However, the material composition and binder contents were different between various construction materials. Therefore, the influence of RTPF on the performance of CPB has remained unclear. The lack of information about the performance and benefit–cost ratio of RTPF-reinforced CPB has been a major inhibiting factor for the application of RTPFs in the field of mine filling. Compared with virgin commercial fibers, recycled fibers are cost-effective with a higher utilization rate of raw materials when it serves as reinforcement material [23]. However, the recycled fibers, such as scrap tire rubber fibers, usually had an adverse impact on the mechanical strength of CPB [19]. The utilization of RTPF for CPB reinforcement that we proposed improved both the mechanical strength and the toughness of the ordinary CPB and increased the cost–benefit ratio of the fiber-reinforced CPB. The main purpose of this study is to explore the effects of RTPFs on the flowability, mechanical properties and failure modes of CPB. The results could provide a reference for the utilization of RTPFs in CPB technology, which could contribute to the reduction in carbon emission with enhanced safety in mining operations.

## 2. Materials and Methods

### 2.1. Materials

#### 2.1.1. Binder, Water and Tailings

**Figure 1:**
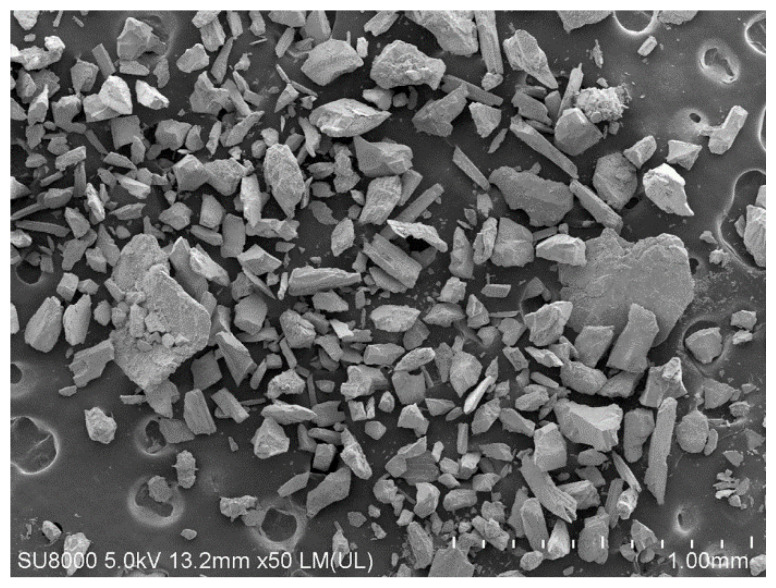
SEM micrograph of the tailings with 50× magnification.

**Figure 2:**
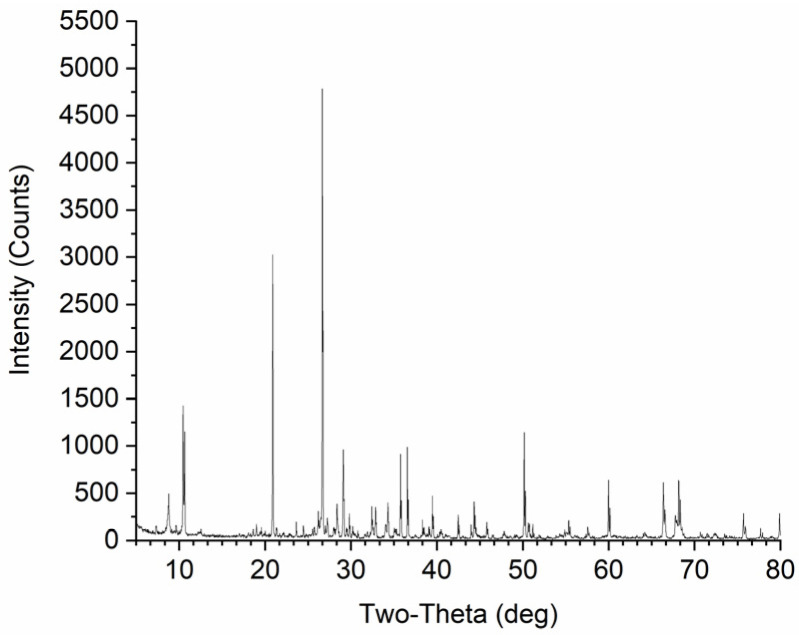
XRD mineralogical composition analysis results of the tailings.

**Figure 3:**
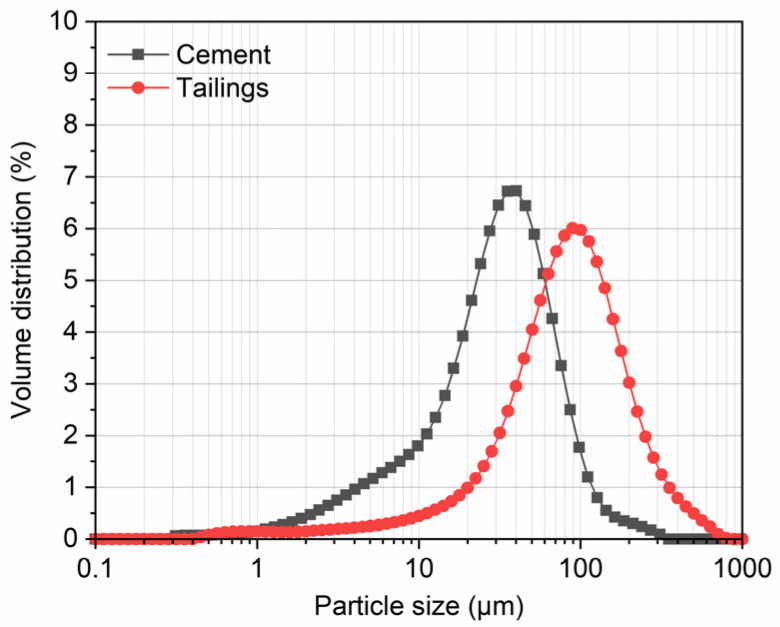
Particle size distribution of the tailings and cement.

Ordinary Portland cement P.O. 42.5 was used as the binder. The most common cement dosage in CPB is between 3% and 10% by the weight of the tailings [14,15,24]. Based on experience from the iron mine, the cement content was chosen to be 5% by dry solids weight in this study. Tap water was used as the mixing water to prepare all CPB specimens [25]. The tailings were sourced from an iron mine located in the southern part of Shandong, China. Figure 1 shows an SEM micrograph of the tailings, which shows the irregular shape of the tailings particles with different sizes. The mineralogical composition of the tailings is shown in Figure 2. A laser particle size analyzer (Malvern Mastersizer 3000, London, UK) was used to determine the particle distribution of the cement and tailings, their results are shown in Figure 3. The main chemical and physical properties of the tailings and cement used in this study are shown in Table 1.

#### 2.1.2. Fibers

**Figure 4:**
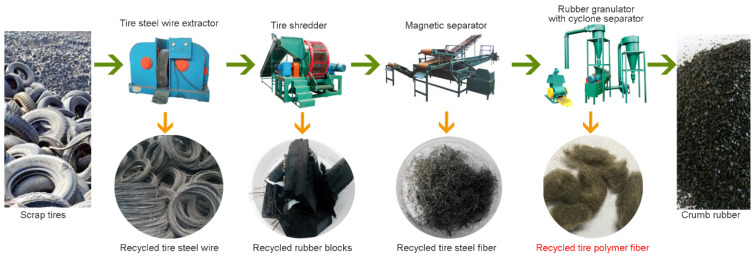
The sketch of the scrap tire recycling process.

**Figure 5:**
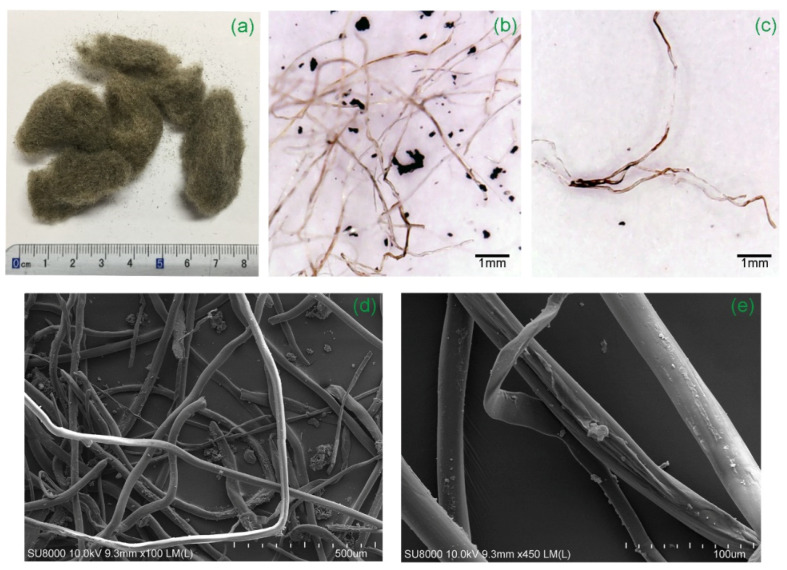
Pictures of RTPFs at (**a**) 1×, (**b**,**c**) 15×, (**d**) 100× and (**e**) 450× magnification.

**Figure 6:**
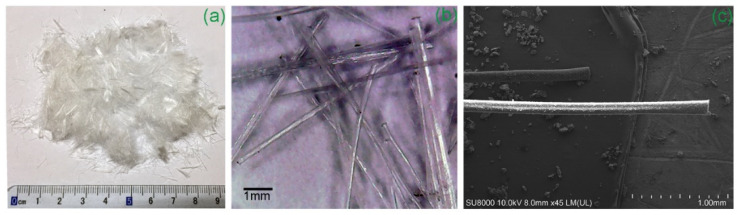
Pictures of CPPFs at (**a**) 1×, (**b**) 15× and (**c**) 45× magnification.

The main characteristics of RTPFs and CPPFs are summarized in Table 2. The RTPFs used in this study refer to a polymer fiber fluff which was a byproduct of the grinding of scrap tires (Figure 4). The average length and diameter of RTPFs were 9.0 mm and 0.03 mm, respectively. As shown in Figure 4, RTPFs were collected using air separators. The RTPF predominantly consists of polyester fibers. Meanwhile, RTPFs could inevitably contain some rubber ash. Figure 5 shows a series of images of RTPFs at different magnifications. It can be seen that most fibers were twisted with traces of surface damage. This may be due to the mechanical damage effect during the grinding process of scrap tire rubber. The CPPF used in this study, as shown in Figure 6, is a kind of commercial virgin material. It can be seen from Figure 6 that CPPFs had good surface quality and were kept in a straight shape. The average length and width of the CPPFs were 6.0 mm and 0.1 mm, respectively. According to accumulated experience [15,26,27], the fiber contents used in this investigation were 0%, 0.3%, 0.6% and 0.9% by dry solids weight.

### 2.2. Preparation of Mixtures and Specimens

CPB mixtures and specimens with varying contents of RTPFs and CPPFs were prepared. The binder content and the solid content of the mixtures were determined as 5% and 75%, respectively. In order to prepare CPB specimens precisely, the materials were weighed on an electric scale with an accuracy of 0.01 g. The dry tailings with specific amounts of cement and tap water were initially mixed in a laboratory mixer while the pre-weighed amount of RTPFs or CPPFs was added. The mixing time for all mixtures was kept constant at 15 min [27]. Once the mixing time was reached, the mixture was cast into cylindrical molds of 50 mm in diameter and 100 mm in height to prepare CPB specimens. The specimens were cured at room temperature for 12 h before the demolding. After the demolding, the specimens were transferred into a humidity chamber at a temperature of 20 °C and relative humidity of 95% for 7 days or 28 days of curing. At least two specimens for each mixing ratio were prepared for the following experimental tests to ensure the repeatability of the results [28].

### 2.3. Testing Methods

#### 2.3.1. Slump Test

**Figure 7:**
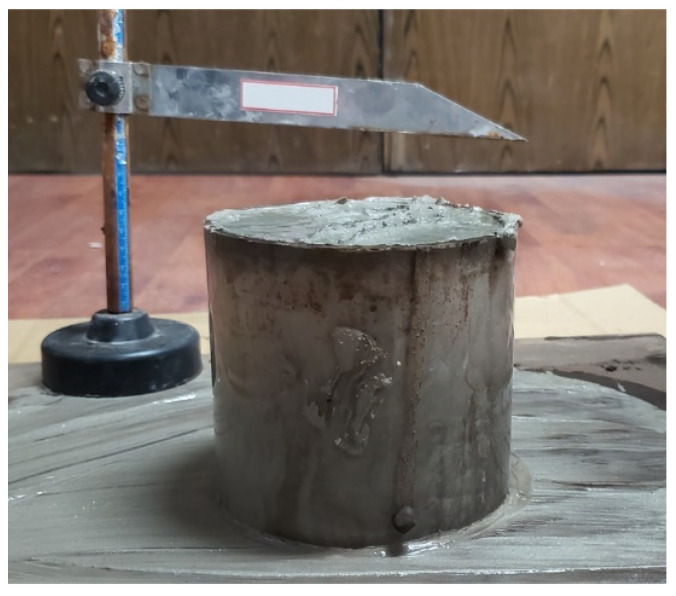
Pictorial view of the cylindrical slump test device in this study.

The slump test has become a widely used testing method to evaluate the flowability of CPB [25,29,30]. Researchers are more likely to choose the ASTM C 143 standard [31] as the reference for the CPB slump tests. However, due to the much smaller particle size and lower solid content of CPB compared with concrete, the standard ASTM C 143 might not be suitable for CPB perfectly. In 2018, Niroshan et al. [32] recommended a cylindrical slump test method for CPB, the test device was a smaller cylinder (110 mm in diameter and 110 mm in height) as shown in Figure 7. In this study, the slump test was conducted using the cylindrical slump test device based on the experience of the previous literature [32].

#### 2.3.2. Unconfined Compression Test

It is generally believed that the unconfined compression test results can give information about the unconfined compressive strength (UCS) and stress-strain relations of the investigated material [27,29,30]. In this investigation, the unconfined compression test was carried out based on accumulated experience [15,16] and ASTM C39 [33] guidelines. A computer-controlled mechanical press system was used for carrying out the tests. The cured specimen was placed axially between the two bearing plates and loaded at a constant displacement rate of 0.2 mm/min until complete failure [19]. The loading data, including force and displacement, were recorded by a data acquisition and processing system and saved by the computer controlled system. A digital camera was used to photograph the failure process of the tested specimens.

#### 2.3.3. Scanning Electron Microscopy (SEM) Analysis

SEM was carried out to investigate the microstructure of the fiber and fiber reinforced CPB. A field emission gun scanning electron microscope (Hitachi SU8000, Tokyo, Japan) was used for the SEM analysis. Fibers were dispersed and spread evenly on the top of the objective table. The cured specimen was cut into some 2 mm thick pieces and coated with platinum.

## 3. Results and Discussion

### 3.1. Flowability of Fresh CPB Slurry

**Figure 8:**
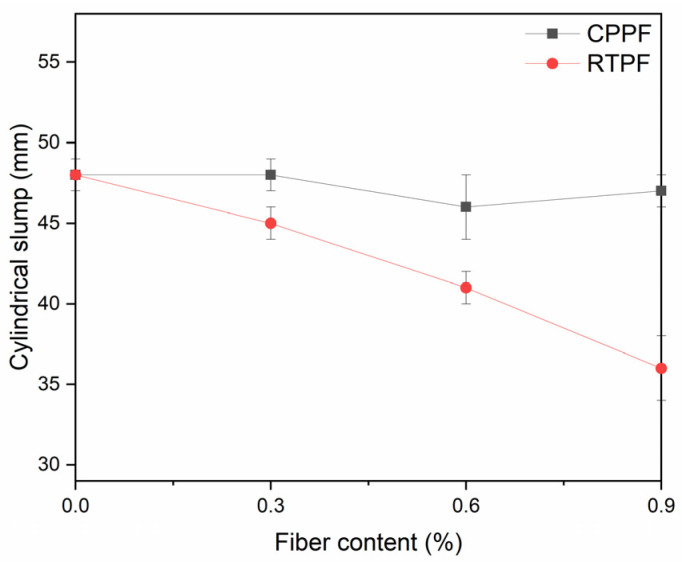
Effects of fiber content on the cylindrical and standard slump values of CPB mixtures.

Cylindrical slump values of fiber-reinforced CPB slurries are shown in Figure 8, where the error bars show the standard deviation. It can be seen that the increase of RTPF content reduces the slump value of CPB, while CPPF content had little impact on it. It may be explained by the fact that the CPPF had a very low moisture regain of 0.03%, while the RTPF had a higher moisture regain of 0.4%. In addition, there was some rubber ash in the RTPFs, which had a water absorption of 5%. Therefore, the increase of RTPF content may lead to the reduction in free water in CPB mixtures, which may be responsible for the decrease in slump values. On the other hand, twisted RTPFs were easy to get tangled and form a reticular structure, which could help maintain the shape of the CPB mixture pile. According to the relation equation of cylindrical slump and standard slump [32], it can be found that the RTPF-reinforced CPB had a good flowability (more than 40 mm) when the fiber content was limited to less than 0.6%.

### 3.2. Mechanical Properties

**Figure 9:**
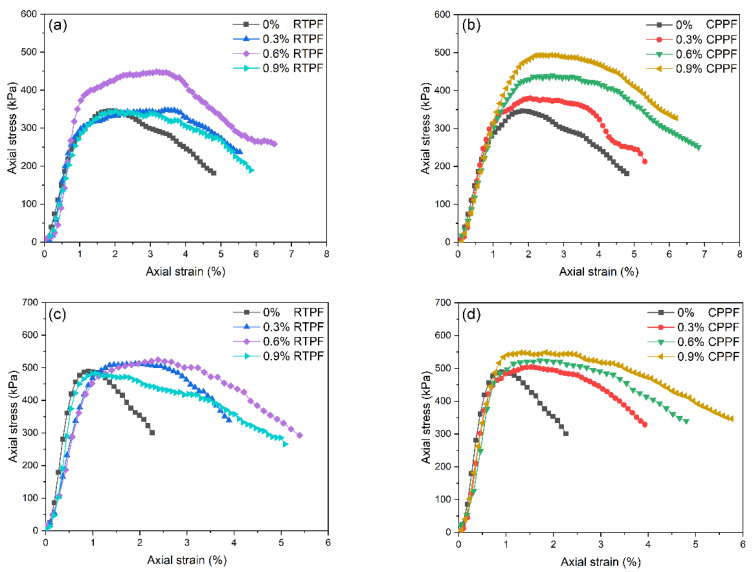
Stress–strain curves of the 7-day-cured (**a**) RTPF-reinforced CPB, (**b**) CPPF-reinforced CPB and 28-day-cured (**c**) RTPF-reinforced CPB and (**d**) CPPF-reinforced CPB.

After processing the experimental data, typical stress–strain curves of the investigated CPB are shown in Figure 9. It can be seen that the stress of the ordinary CPB decreased sharply after attaining the peak stress with a small failure strain, which indicated that the stress-strain curve tends to exhibit a brittle compressive behavior. The brittle behavior of the CPB was more pronounced at 28 days curing age than at 7 days curing age. It can also be seen preliminarily that the incorporation of fibers had a limited effect on the elastic modulus of CPB, but it could significantly change the stress–strain relationship at the plastic stage. This finding reflects those of Yi et al. [15], who also found the effects of fibers on the change of plastic stage. Compared with the ordinary CPB specimen, the fiber-reinforced CPB specimen seemed to have a larger failure strain and a more ductile compressive behavior. For further investigation, some mechanical performance indexes were utilized to quantify the compressive behavior of the investigated CPB. The effects of RTPFs and CPPFs on the failure strain, UCS and toughness are discussed in the following sections.

#### 3.2.1. Failure Strain

**Figure 10:**
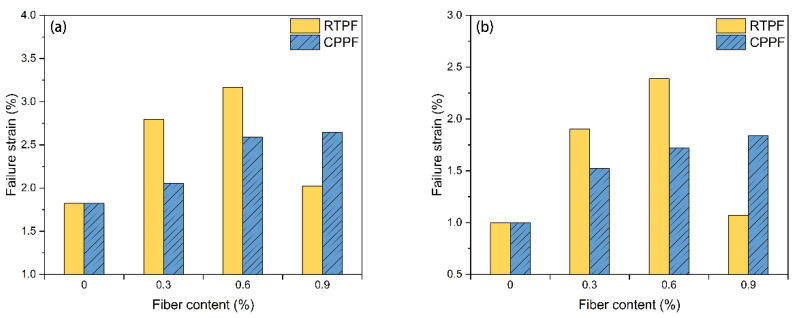
Failure strains of (**a**) 7-day-cured CPB and (**b**) 28-day-cured CPB.

**Figure 11:**
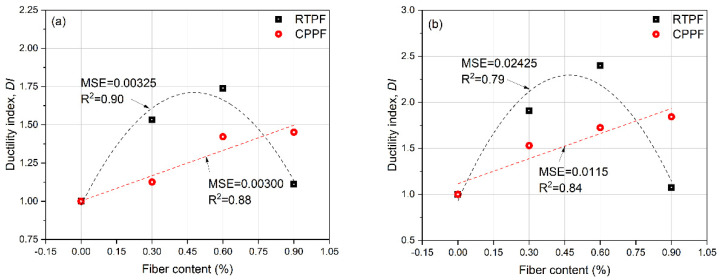
Ductility indexes of (**a**) 7−day−cured CPB and (**b**) 28−day−cured CPB.

Failure strain refers to the axial strain at peak stress. It has been regarded as an important mechanical parameter that reflects the ductility of CPB [34]. Figure 10 summarizes the failure strain values of CPB with different fiber contents and curing ages. It was observed that the fiber-reinforced CPB had a larger failure strain than the ordinary CPB. Moreover, the failure strain increased with the increasing fiber content for both curing ages. The only exception was the RTPF-reinforced CPB with 0.9% fiber content, which showed a decrease in the failure strain. These results indicate that the increase in CPPF content increased the failure strain of fiber-reinforced CPB, while RTPFs could maximize the failure strain of fiber-reinforced CPB when the fiber content was around 0.6%. It can be noted that after the introduction of fibers, the improvement of failure strain varied between different curing ages and fiber types. To reveal the improvement effect of fibers more intuitively, a ductility index (*DI*) was proposed as follows to quantify the improvement of fibers on the ductility of CPB:

(1) $$DI=\frac{\Delta_{fiber}}{\Delta_{nofiber}}$$

where $\Delta_{fiber}$ and $\Delta_{nofiber}$ are, respectively, the failure strains of the fiber-reinforced CPB and the ordinary CPB at the same curing age and same cement content. The results shown in Figure 11 exhibited that when the fiber content increased, the CPPF-reinforced CPB ductility index increased gradually, whereas the RTPF-reinforced CPB ductility index increased first and then decreased. The maximum *DI* of the CPPF and RTPF were achieved when the fiber contents were 0.9% and 0.6%, respectively. For CPB specimens with 7-day and 28-day curing ages, these trends were basically the same.

#### 3.2.2. Unconfined Compressive Strength

**Figure 12:**
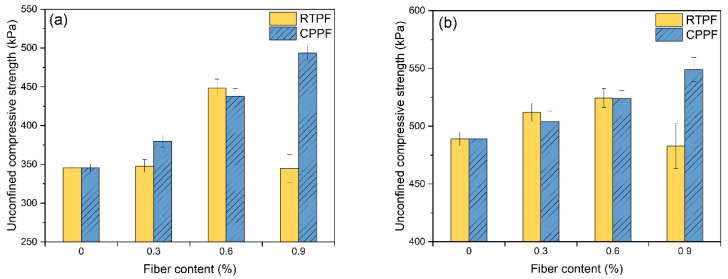
UCS values of (**a**) 7-day-cured CPB and (**b**) 28-day-cured CPB.

**Figure 13:**
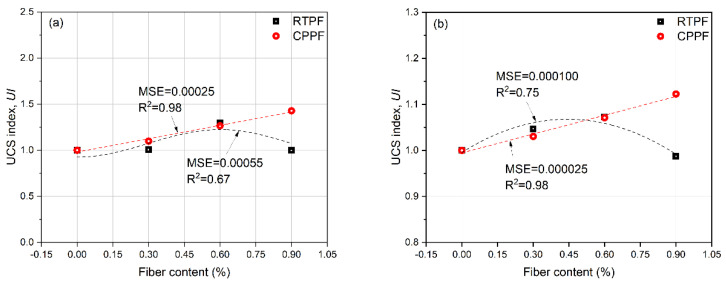
UCS indexes of (**a**) 7−day−cured CPB and (**b**) 28−day−cured CPB.

Figure 12 summarizes the UCS of CPB with different fiber contents and curing ages, where error bars represent the standard deviation. It can be seen that the fiber-reinforced CPB generally had higher UCS compared with the ordinary CPB. After the mixture of 0.9% CPPFs, the UCS of 7−day−cured CPB increased from 345.8 kPa to 493.7 kPa, and the UCS of 28−day−cured CPB increased from 489.1 kPa to 549.0 kPa. This indicated the stable improvement effect of the CPPF as an acclaimed virgin commercial fiber. It can also be seen that UCS of the RTPF-reinforced CPB increases first and then decreases with the increase in fiber content in the investigated range. The highest UCS values of the RTPF-reinforced CPB specimens at 7−day and 28−day curing ages were found to be 448.3 kPa and 524.4 kPa, respectively, when the fiber content is 0.6%. In order to research the improvement effect of fibers on the UCS of CPB, a UCS index (*UI*) was proposed as follows:

(2) $$UI=\frac{\sigma_{fiber}}{\sigma_{nofiber}}$$

where $\sigma_{fiber}$ and $\sigma_{nofiber}$ are, respectively, the UCS values of the fiber-reinforced CPB and the ordinary CPB at the same curing age and same cement content. The results of *UI* are summarized in Figure 13. Unsurprisingly, CPPFs exhibited a stable reinforcement ability with increasing fiber content [16]; the maximum *UI* of the CPPF-reinforced CPB was achieved when the fiber content was 0.9% in this study. While the *UI* of the RTPF-reinforced CPB increased first and then decreased with the increasing fiber content, the maximum *UI* of the RTPF-reinforced CPB was achieved when the fiber content was 0.6% in this study. These results are consistent for specimens with 7-day and 28-day curing ages. 

#### 3.2.3. Toughness

**Figure 14:**
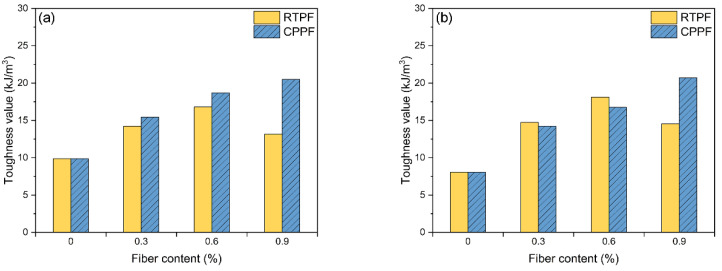
Toughness values of (**a**) 7−day−cured CPB and (**b**) 28−day−cured CPB.

**Figure 15:**
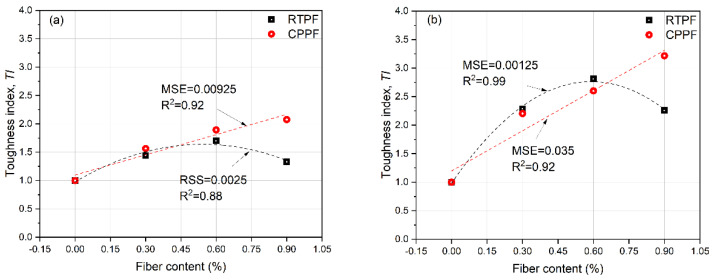
Toughness indexes of (**a**) 7−day−cured CPB and (**b**) 28−day−cured CPB.

Toughness reveals the capability of a material to absorb energy and plastically deform, which is important for the safety and stability of mining work. According to the previous research, the toughness value of CPB can be determined by calculating the area under the stress–strain curve up to the fracture strain [19,35]. It can be seen in Figure 14 that all of the fiber-reinforced CPB had higher toughness values than the ordinary CPB. The toughness value of the CPPF-reinforced CPB increased steadily with the increased fiber content. The toughness value of the RTPF-reinforced CPB increased first and then decreased with increased fiber content. The maximum toughness values of the CPPF-reinforced CPB and RTPF-reinforced CPB were achieved when the fiber contents were 0.9% and 0.6%, respectively. Both the 7-day-cured and 28-day-cured CPB showed a similar trend. For 7-day curing age, the maximum toughness values of the RTPF-reinforced CPB and CPPF-reinforced CPB were 16.79 KJ/m^3^ and 20.50 KJ/m^3^, respectively. For 28-day curing age, the maximum toughness values of the RTPF-reinforced CPB and CPPF-reinforced CPB were 18.12 KJ/m^3^ and 20.72 KJ/m^3^, respectively. In order to research the improvement effect of fibers on the toughness of CPB, a toughness index (*TI*) was proposed as follows:

(3) $$TI=\frac{\varepsilon_{fiber}}{\varepsilon_{nofiber}}$$

where $\varepsilon_{fiber}$ and $\varepsilon_{nofiber}$ are, respectively, the toughness values of the fiber-reinforced CPB and the ordinary CPB at the same curing age and same cement content. The results of *TI* are summarized in Figure 15. It can be seen that CPPF performed a stable reinforcement ability with the increasing fiber content: the maximum *TI* of the CPPF-reinforced CPB of 7-day and 28-day curing ages were 2.08 and 3.22, respectively. They were achieved when the CPPF content was 0.9%. While *TI* of the RTPF-reinforced CPB increased first and then decreased with the increasing fiber content, the maximum *TI* of the RTPF-reinforced CPB of 7-day and 28-day curing ages were 1.70 and 2.81, respectively. They were achieved when the RTPF content was 0.6%. These results echo those of *UI* and *DI*, which indicate that the toughness of CPB can be enhanced significantly by adding appropriate amounts of RTPFs. It was noted that the 28-day-cured fiber-reinforced CPB achieved higher *TI* than the 7-day-cured fiber-reinforced CPB, which may be responsible for the hydration products produced during the longer curing time.

These results confirm that the inclusion of fiber can improve the mechanical properties of CPB, which is in agreement with the previous research [16,27]. However, in this study, we found that the method of simply increasing the fiber content to further improve the mechanical properties of fiber-reinforced CPB may not be applicable for the RTPF-reinforced CPB. When the RTPF content was increased from 0.6% to 0.9%, the mechanical properties of CPB did not continue to improve but instead showed a decrease. The deterioration mechanisms of specimens with 0.9% of RTPF fibers were summarized as follows. On the one hand, twisted RTPFs could be tangled up naturally (as shown in Figure 5). As the fiber content increased, the interaction between fibers increased. Beyond a certain fiber content, the interaction among fibers could substantially increase and fiber clumping may occur [36]. Fiber clumping may lead to the non-uniform distribution of fibers in CPB and decrease the mechanical properties of CPB significantly. On the other hand, unlike virgin commercial fibers, recycled fibers contained impurities that were primarily rubber ash, as mentioned in Section 2.1.2. The addition of rubber ash in the cement-based materials could decrease the mechanical properties [37,38]. In conclusion, when the RTPF content was within 0.6%, the reinforcing effect of the fiber could offset the degradation effect of the rubber ash, and the mechanical properties of CPB were significantly improved. However, when the RTPF content continued to increase to 0.9%, the fibers could not produce effective reinforcement due to fiber clumping, which may also lead to the inhomogeneity of the specimens, and the degradation of the rubber ash was relatively more obvious, resulting in the degradation of the mechanical properties of the RTPF-reinforced CPB. Therefore, it is important to determine the optimal fiber content of RTPFs to achieve its best improvement effect on CPB. 

### 3.3. Failure Modes

**Figure 16:**
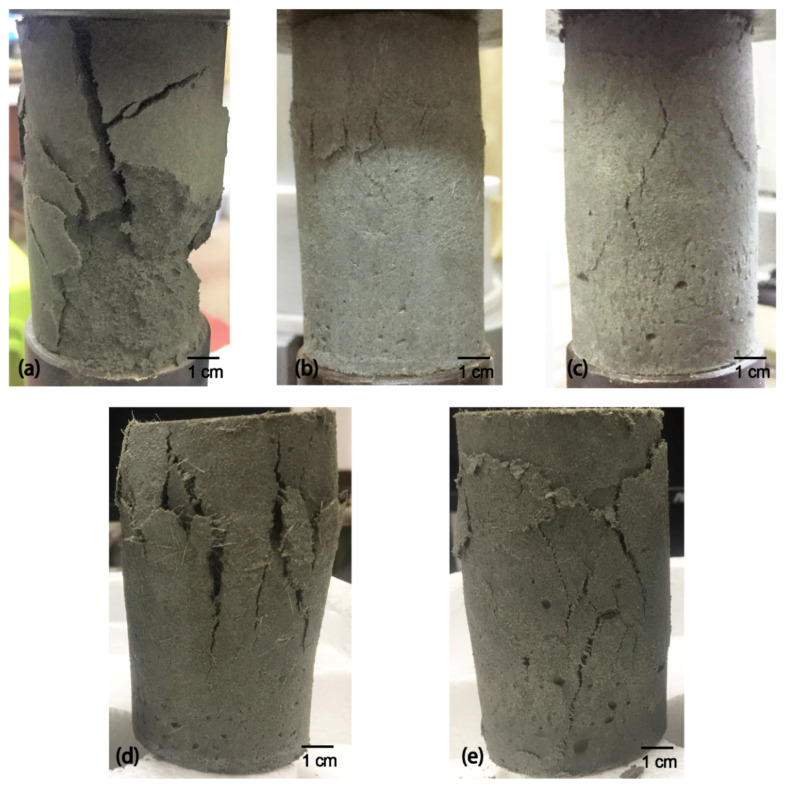
Typical failure modes of the 28−day−cured (**a**) ordinary CPB under 2.5% strain, (**b**) 0.6% CPPF-reinforced CPB under 2.5% strain, (**c**) 0.6% RTPF-reinforced CPB under 2.5% strain, (**d**) 0.6% CPPF-reinforced CPB under 5% strain and (**e**) 0.6% RTPF-reinforced CPB under 5% strain.

The typical failure modes of the ordinary CPB, CPPF-reinforced CPB and RTPF-reinforced CPB are shown in Figure 16. It can be seen from Figure 16a that the ordinary CPB showed one or two major wide cracks with a big mutilation caused by the falling of big blocks under 2.5% strain. The sudden failure of the ordinary CPB structure with the falling of big blocks may cause severe accidents such as smashing and injuring staff without any warning or response time. When under the same strain, it can be seen from Figure 16b,c that the CPPF and RTPF-reinforced CPB only showed several sporadic minor cracks. This echoed the relatively larger failure strains of the fiber-reinforced CPB specimens. It can be seen from Figure 16d that the CPPF showed several obvious small cracks and finally failed under 5% strain. The CPPF-reinforced specimen had exhibited obvious bulging failure and there were some small fragments. The RTPF-reinforced CPB showed a similar failure mode to the CPPF-reinforced CPB as shown in Figure 16e; several small cracks and some falling debris were found, but there was no falling blocks and the specimen just remained integrated, even under 5% strain. This may be benefitial for improving the safety of mining work [15]. In the area with frequent geostress activity, the rock stratum may occur large deformations suddenly [39]. The RTPF-reinforced CPB would remain integrated without blocks falling under a large strain, which provides precious response time for evacuations or temporary reinforcement and could avoid safety accidents involving people being injured by big falling CPB blocks.

### 3.4. Microscopic Observations

**Figure 17:**
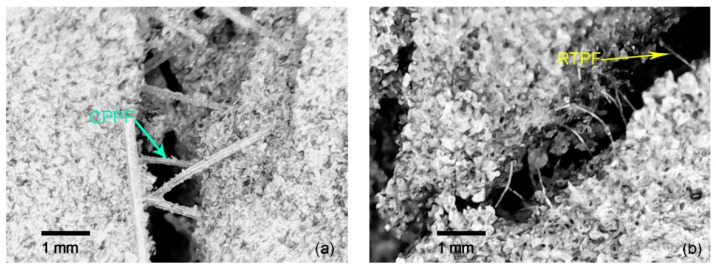
Microscope photos at 25× magnification of (**a**) the CPPF-reinforced CPB and (**b**) the RTPF-reinforced CPB.

When cracks developed during the compression of CPB, fibers could bridge these cracks and prevent the further development of cracks [27]. Both RTPFs and CPPFs were found to be the bridge of the crack in CPB matrix, as shown in Figure 17. This indicated that RTPF had the same good bridging ability as CPPF. However, when we checked specimens and took these pictures, it was noted that more cracks without RTPF bridging could be found in the 0.9% RTPF-reinforced CPB specimen than in the CPPF-reinforced CPB specimen. This may echo the uneven distribution of RTPF when the fiber content was 0.9%, mentioned in Section 3.2.3. Therefore, the uniform distribution of fibers may be a prerequisite for the fiber bridging effect to act as a structural improvement.

**Figure 18:**
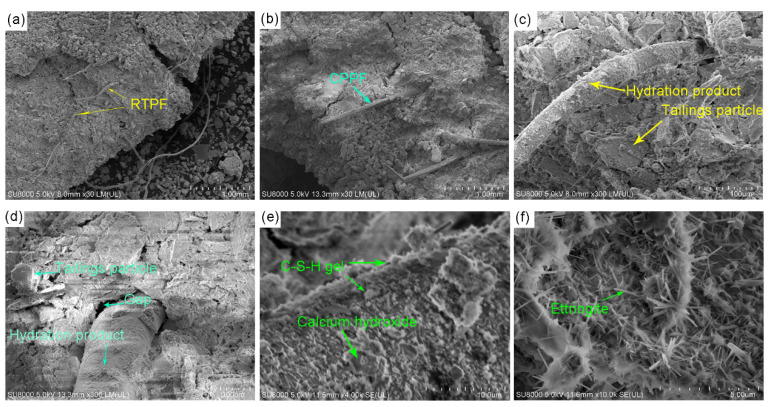
SEM images of (**a**) RTPF-reinforced CPB and (**b**) CPPF-reinforced CPB at 30× magnification, (**c**) RTPF and (**d**) CPPF at 300× magnification, (**e**) hydration products at 4000× magnification and (**f**) hydration products at 10,000× magnification.

Figure 18 shows SEM images of 28-day-cured fiber-reinforced CPB with a 0.6% fiber content. As shown in Figure 18a, twisted RTPFs can be easily found in the CPB matrix. Unlike RTPFs, CPPFs kept their straight shape in the CPB matrix as shown in Figure 18b. Tailings particles in the CPB matrix can be found in Figure 18c. This may have been due to the fact that iron tailings was inert at ambient temperature with almost no pozzolanic activity [40]. Tailings would be the aggregate of CPB matrix and act as a skeleton. In Figure 18d, a small gap may be found between the fiber and CPB matrix, which demonstrated that the interface between dissimilar materials may be a weak link. As shown in Figure 18c–f, plenty of hydration products could be found on the RTPF and CPPF surface in the CPB, which exhibited a good hydration effect. The hydration products may increase the friction between the fiber and CPB matrix, thus helping improve the bridging effect. Future research is suggested to focus on the bonding effect between the fiber and CPB matrix to improve the mechanical performance of fiber reinforced CPB.

### 3.5. Cost–Benefit Analysis

A cost–benefit analysis is a classic method of economics, which compares the estimated costs and benefits to measure the benefits of a decision [41]. Traditional cost and benefit analysis involves all costs of a project and subtracting (or dividing) that amount from the total benefits of the project. In this study, a modified cost–benefit analysis was proposed to be the method of measuring the economic efficiency of using the RTPF-reinforced CPB. According to the previous research on the cost–benefit analysis of new construction material, the increased material cost and the gained mechanical properties were considered for calculating the cost and benefit [1,30]. In this study, the gained mechanical properties were evaluated by the sum of *DI*, *UI* and *TI* values, and the increased material cost came from fibers. Therefore, the mechanical properties cost–benefit analysis of the RTPF-reinforced CPB can be calculated based on Equation (4) below:

(4) $$G_{i}=\frac{C_{0}\left(D_{i}+U_{i}+T_{i}\right)}{C_{i}\left(D_{0}+U_{0}+T_{0}\right)}$$

where *i* represents the fiber content (%) of the CPB, *G_i_* is the gained mechanical properties, *C_i_* is the increased material cost and *D_i_*, *U_i_*, and *T_i_* are the values of *DI*, *UI*, and *TI* of the CPB, respectively. In order to give a wider reference significance, we performed a non-dimensional treatment for the material costs. Based on the field investigation, the RTPF: CPPF price ratio was approximately 1:1.8. Let the *C*_0_ be one, and the unit price of the RTPF be one. The material cost (*C_i_*) was calculated by the sum of the ordinary CPB cost (*C_0_*) and the fiber cost (fiber unit price times fiber content).

**Figure 19:**
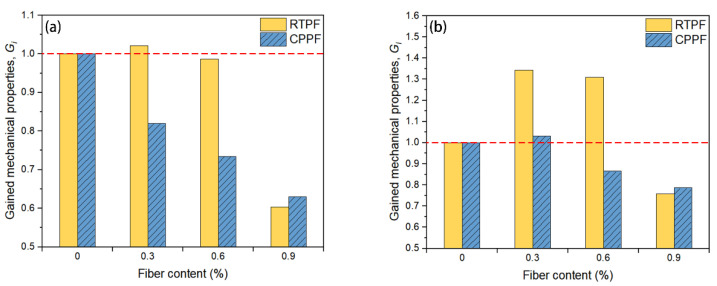
Cost–benefit analysis results of fiber reinforced CPB at (**a**) 7-day-curing age and (**b**) 28-day-curing age.

The *G_i_* of the RTPF-reinforced CPB and CPPF-reinforced CPB with different fiber content can be calculated based on Equation (4), respectively. The calculated results are summarized in Figure 19. A red horizontal line (*G_i_* = 1.0) was set to be the datum line of the cost–benefit analysis. Columns above the datum line indicate that the corresponding fiber-reinforced CPB has a better benefit–cost ratio; to be more specific, it gains better mechanical properties with lower material costs. In contrast, columns below the datum line indicate that the corresponding fiber-reinforced CPB has a worse benefit–cost ratio. It can be seen from Figure 19 that *G_i_* values of the RTPF-reinforced CPB were generally higher than those of the CPPF-reinforced CPB, except when the fiber content was 0.9%. This indicated that the inclusion of appropriate amounts of RTPFs into CPB could gain relatively high mechanical properties with a lower material cost than the inclusion of CPPFs. This may be due to the unit price of the RTPF being lower than that of the CPPF, and the reinforcement effect of RTPFs and CPPFs on the mechanical properties were similar. It can be noticed that *G_i_* values decreased with the increase in fiber content both for CPPFs and RTPF-reinforced CPB when the fiber content exceeded 0.3%, which may have indicated that the increased material costs of further increasing fiber content outweighed the gain on the mechanical properties. Thus, there would be an optimal fiber content for the RTPF-reinforced CPB that could obtain relatively high mechanical properties with the lowest material costs. It can also be seen from Figure 19a that *G_i_* values were generally low for the 7-day-cured fiber-reinforced CPB. Only the 7-day-cured RTPF-reinforced CPB with 0.3% fiber content had a higher *G_i_* than the datum line, which was 1.02. Figure 19b shows that the 28-day-cured RTPF-reinforced CPB with fiber contents of 0.3% and 0.6% achieved higher *G_i_* values of 1.34 and 1.31, respectively. The *G_i_* of the CPPF-reinforced CPB with 0.3% fiber content was 1.03, which marginally outstripped the datum line. This may have been due to the fact that a longer curing age allowed sufficient hydration reaction in the CPB, which could contribute to a better bond between fibers and the CPB matrix, thus improving the reinforcement effect of fibers while keeping material costs constant. These results indicate that using RTPFs to reinforce CPB has a good benefit–cost ratio.

## 4. Conclusions

This study investigated the feasibility of using RTPFs to reinforce CPB. For comparative purposes, the performance of the CPPF-reinforced CPB was also investigated. Based on the investigation, the following conclusions could be drawn:

- The increase in RTPF content could decrease the flowability of CPB, while CPPFs have little impact on it. The RTPF-reinforced CPB had a good flowability (more than 40 mm) when the fiber content was limited to less than 0.6%.
- The inclusion of RTPFs or CPPFs improves the failure strain, UCS and toughness of CPB. Although increasing the CPPF content can continuously improve the mechanical properties of CPB, optimal fiber content of the RTPF is identified for the best compressive mechanical property of the RTPF-reinforced CPB. In this study, the optimal fiber content of RTPF is 0.6%.
- The ordinary CPB showed a brittle failure with wide major cracks and falling blocks, while the RTPF-reinforced CPB showed a bulging failure mode with several small cracks. The RTPF-reinforced CPB could remain integrated under a large strain. This is important for avoiding the sudden structure failure and falling of big blocks, resulting in reduced injuries. Microscopic observations of the fiber-reinforced CPB showed that the bridge effect of RTPFs and CPPFs is responsible for preventing the development of cracks and enhancing structural strength if the fibers are uniformly distributed.
- The inclusion of appropriate amounts of RTPFs into CPB could gain relatively high mechanical properties with a lower material cost. Due to the better bond between fibers and the CPB matrix, the *G_i_* value of RTPF-reinforced CPB at 28-day-curing age was higher than that at 7-day-curing age.

These findings indicate that the RTPF-reinforced CPB with an optimal fiber content had similar mechanical properties to the CPPF-reinforced CPB. Better still, the material cost of the RTPF-reinforced CPB is lower while its environmental benefit is higher. 

Future studies could focus on improving the fiber distribution and bonding effect between the fiber and CPB matrix using better mixing and bonding technology.

## Figures and Tables

**Table 1:** Main chemical and physical properties of the tailings and cement.

Chemical Composition	Tailings	Cement	Physical Properties	Tailings	Cement
SiO_2_	55.50	21.40	Specific gravity	2.76	3.10
Al_2_O_3_	2.93	4.31	Specific surface (cm^2^/g)	2640	3580
Fe_2_O_3_	23.80	4.91	*D*_10_ (μm)	20.41	6.66
MgO	3.18	3.00	*D*_50_ (μm)	79.62	33.2
CaO	5.26	62.34	*D*_90_ (μm)	208.89	81.2
SO_3_	0.41	2.20	-	-	-
Na_2_O	0.62	-	-	-	-
K_2_O	0.80	-	-	-	-
P_2_O_5_	0.38	-	-	-	-
MnO	0.21	-	-	-	-
TiO_2_	0.12	-	-	-	-

**Table 2:** The main characteristics of the RTPF and CPPF.

Physical Properties	RTPF	CPPF	Other Features	RTPF	CPPF
Specific gravity	0.96	0.91	Major ingredient	Polyester	Poly-propylene
Average length (mm)	10.0	6.0	Impurity	Rubber	None
Average diameter (mm)	0.03	0.1	-	-	-
Moisture regain (%)	>0.4	<0.03			
Tensile strength (MPa)	620	590	-	-	-

## Data Availability

The datasets used or analyzed during the current study are available from the corresponding author upon reasonable request.
